# Effects of ischemic postconditioning and long non-coding RNAs in ischemic stroke

**DOI:** 10.1080/21655979.2022.2108266

**Published:** 2022-11-24

**Authors:** Wei Ma, Kewei Zhu, Luwei Yin, Jinwei Yang, Jinfen Zhang, Hongjie Wu, Kuangpin Liu, Chunyan Li, Wei Liu, Jianhui Guo, Liyan Li

**Affiliations:** a Institute of Neuroscience, Faculty of Basic Medical Science, Kunming Medical University, Kunming, China; b Second Department of General Surgery, First People’s Hospital of Yunnan Province, Kunming, China

**Keywords:** Ischemic stroke, ischemic postconditioning, remote ischemic postconditioning, long non-coding RNAs, neuroprotective effect

## Abstract

Stroke is a main cause of disability and death among adults in China, and acute ischemic stroke accounts for 80% of cases. The key to ischemic stroke treatment is to recanalize the blocked blood vessels. However, more than 90% of patients cannot receive effective treatment within an appropriate time, and delayed recanalization of blood vessels causes reperfusion injury. Recent research has revealed that ischemic postconditioning has a neuroprotective effect on the brain, but the mechanism has not been fully clarified. Long non-coding RNAs (lncRNAs) have previously been associated with ischemic reperfusion injury in ischemic stroke. LncRNAs regulate important cellular and molecular events through a variety of mechanisms, but a comprehensive analysis of potential lncRNAs involved in the brain protection produced by ischemic postconditioning has not been conducted. In this review, we summarize the common mechanisms of cerebral injury in ischemic stroke and the effect of ischemic postconditioning, and we describe the potential mechanisms of some lncRNAs associated with ischemic stroke.

## Graphical abstract

**Figure:**
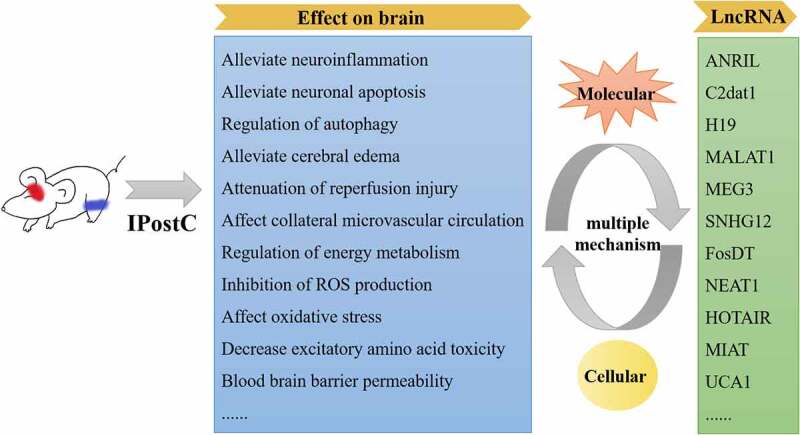

## Introduction

Stroke is the second leading cause of death worldwide [1]. It is an acute disease involving an impaired cerebral blood supply and is characterized by high morbidity, mortality, recurrence rates, disability rates, and economic burden. Acute ischemic strokes caused by intracranial artery occlusion or extracranial carotid artery occlusion account for approximately 85% of all strokes. Cerebral infarction is a disorder of the blood supply to local brain tissue that leads to cerebral ischemia and hypoxia. The mechanism of cerebral ischemic injury is highly complex, and this complexity mainly manifests as inflammatory injury, excitatory amino acid release, intracellular Ca^2+^ overload, free radical formation, protease activation, blood-brain barrier injury, mitochondrial energy disturbance, excessive nitric oxide (NO) levels, excitatory amino acid toxicity, and the formation of glial scars (Table 1). This series of pathological and physiological processes interacts with changes in the temporal and spatial molecular mechanisms caused by cerebral ischemic injury. Hypoxia, neuronal necrosis, and an intense inflammatory response are modulated by many biological processes in ischemic stroke [2,3]. Long non-coding RNAs (lncRNAs) play an important role in cell differentiation, apoptosis, and regulation of gene expression under physiological and pathological conditions. In recent years, an increasing number of studies have focused on the important role of lncRNAs in ischemic stroke and their potential as therapeutic approaches. The effect and mechanism of ischemic postconditioning (IPostC) in ischemic stroke and the potential lncRNAs involved in the physiological and pathological processes of ischemic stroke have not been fully clarified. In this review, we summarize the common mechanisms of cerebral injury in ischemic stroke and the effect of IPostC, and we describe the potential association of lncRNAs with neuronal injury and protective mechanisms in ischemic stroke.

**Table 1.** Common mechanisms of cerebral injury in ischemic stroke.

Effect on the brain	Related factors	Mechanisms related to cerebral injury	References
Neuroinflammation	1. Pro-inflammatory cytokines: IL-6, IL-8, IL-1β, COX-2, TNF-α; Anti-inflammatory factors: IL-4 and IL-10 2. MAPK, NLRP inflammasome 3. MiRNA, caspase-3 4. ACSL4, rhFGF21, NF-κB, PPAR-γ	1.Regulation of pro-inflammatory and anti-inflammatory cytokines in the ischemic brain. 2. Activation of the MAPK signaling pathway contributes to activation of the NLRP1 and NLRP3 inflammasome proteins in neurons and brain tissues. 3. Circulating miRNA-3552 interacts with caspase-3 to participate in neuroinflammatory responses. 4. ACSL4 and rhFGF21 modulate microglia/macrophage-mediated neuroinflammation via the NF-κB and PPAR-γ signaling pathways.	1. [5–8,15,16] 2. [9,10] 3. [20] 4. [25]
Neuronal apoptosis	Caspase family, TNF-α, TNF-R, Bcl-2 family, CREB, Hes1, FoxP2, p38 MAPK signaling pathway	Regulation of neuronal cell apoptosis in the ischemic brain.	[18–20]
Steady-state balance of Ca^2+^	NMDA receptor	Activation of NMDA receptors may result in Ca^2+^ influx in neurons and activation of Ca^2+^-dependent protease-induced cell death pathways, resulting in neuronal apoptosis after cerebral ischemia.	[21–23]
Oxidative stress	1. ROS 2. Glutathione peroxidase	1. Oxidative stress induced by ROS overproduction is associated with ischemia-reperfusion injury, neuronal apoptosis, inflammatory signaling pathway activation, and blood-brain barrier damage in ischemic stroke. 2. Neuronal glutathione reduction is the result of oxidative stress in the brain caused by ischemia-reperfusion.	[26–29,139]
Autophagy	1. Beclin-1 2. Malat1 3. MEG3	1. Beclin-1 induces autophagy and regulates the survival or death of ischemic neurons. 2. Downregulation of Malat1 inhibits Beclin1-dependent autophagy in ischemic stroke. 3. MEG3 is upregulated and interacts with p53 to mediate neuron autophagy in ischemic stroke.	1. [20] 2. [30] 3. [31]
Energy metabolism disorders	1. NADPH 2. PGC-1α 3. AMPK	1. NADPH diaphorease expression is upregulated in hippocampal astrocytes after ischemic stroke. 2. PGC-1α increases mitochondrial fatty acid oxidation gene expression in ischemic brain tissue. 3. AMPK resists cerebral ischemic injury by activating catabolic pathways and inactivating ATP-consuming processes.	1. [33] 2. [35] 3. [36]
Excessive intracellular free radicals and NO	1. Superoxide dismutase 2. Nitric oxide synthase	1. Superoxide dismutase protects the brain by removing excessive free radicals from cells. 2. Increases in nitric oxide synthase expression and NO production by reactive astrocytes may play a role in delayed neuronal death after ischemic damage.	1. [37] 2. [11]
Excitatory amino acid toxicity	1. Glutamate levels, glutamate transporters (glt-1), gap junction proteins (Cx43), Na^+^-K^+^-ATPase activity 2. NMDA receptors 3. NO, oxygen free radicals	1. The expression levels of glutamate, glt-1, Cx43, and AMPARs and Na^+^-K^+^-ATPase activity are related to excitotoxicity and anti-excitotoxicity in ischemic stroke. 2. NMDA receptor antagonists can inhibit the release of excitatory amino acids during ischemic stroke. 3. The neurotoxicity of NO is mainly caused by its reaction with oxygen free radicals.	1. [39,40] 2. [41,42] 3. [43]
Blood-brain barrier damage	1. Aquaporin-4 2. Mitochondrial proteins 3. A Na^+^ channel blocker and an antioxidant: AM-36 4. MMP-2/MMP-9	1. Downregulation of aquaporin-4 can increase the integrity of the blood-brain barrier and improve cerebral blood flow after transient MCAO. 2. Nitrite may increase cerebral blood flow via its effect on nitrosylation of mitochondrial proteins. 3. AM-36 exhibits a neuroprotective effect by modulating neutrophil accumulation in the ischemic brain. 4. Activation of MMP-2 or MMP-9 contributes to the breakdown of the blood-brain barrier in ischemic stroke. High serum MMP-9 levels are related to an increased risk of death and disability in acute ischemic stroke.	1. [45] 2. [46] 3. [47,48] 4. [49–51]
Formation of glial scars	Cytokines from neurons and neuroglial cells, such as vimentin, nestin, IL-1β, monocyte chemotactic protein-1, and glial fibrillary acidic protein	Cytokines contribute to the development of reactive gliosis and the formation of glial scars.	[14,52]

IL: interleukin, COX: cyclooxygenase, TNF: tumor necrosis factor, MAPK: mitogen-activated protein kinase, NLRP: nucleotide-binding oligomerization domain, leucine rich repeat and pyrin domain containing protein, miRNA: microRNA, ACSL: acyl-CoA synthetase long chain, NF-κB: nuclear factor kappa B, PPAR: peroxisome proliferator-activated receptor, NMDA: N-methyl-D-aspartate, ROS: reactive oxygen species, CREB: cAMP-response element binding protein. Malat1: metastasis-associated lung adenocarcinoma transcript 1, MEG3: maternal expression gene 3, NADPH: nicotinamide adenine dinucleotide phosphate, PGC: peroxisome proliferator-activated receptor-gamma coactivator, AMPK: adenosine 5’-monophosphate-activated protein kinase, NO: nitric oxide, MMP: matrix metalloproteinase, MCAO: middle cerebral artery occlusion.

## 1. Common mechanisms of cerebral injury in ischemic stroke

Neuroinflammation: Studies on the role of inflammation in acute ischemic stroke were initially focused on the reperfusion phase. The existence of an inflammatory penumbra allows the diapedesis of T cells into intact brain regions, where they trigger neuronal cell death by at least two mechanisms, direct cytotoxicity by perforin secretion and interferon-γ secretion [4]. Pro-inflammatory and anti-inflammatory factors are associated with inflammatory responses in cerebral ischemic injury. The expression levels of the pro-inflammatory cytokines tumor necrosis factor alpha (TNF-α), interleukin (IL)-6, IL-8, IL-1β, and cyclooxygenase-2 were increased after cerebral ischemia [5–7]. Anti-inflammatory factors, including IL-4 and IL-10, protect the brain from ischemia-reperfusion and inflammation-induced injury. Studies have shown that IL-10 deficiency can exacerbate inflammation in the brain [8]. Activation of the mitogen-activated protein kinase (MAPK) signaling pathway contributes to the activation of the NOD-like receptor protein 1 and 3 inflammasome proteins in neurons in vitro and in mouse brain tissues in vivo following ischemic stroke [9,10]. Neuroinflammation leads to neurodegeneration and brain damage: Neuroinflammation is associated with the activation and release of proinflammatory mediators from resident immune cells in the brain (microglia and astrocytes) and peripherally derived immune cells, such as neutrophils, monocytes, and T cells [11]. Progressive neuroinflammation not only damages brain cells, but also plays a positive role in nerve repair activities after cerebral ischemia. Studies have found that the differentially expressed circulating microRNA (miRNA)-3552 interacts with caspase-3 to participate in neuroinflammatory responses in ischemic stroke [12]. All stages of an ischemic episode, from cessation of cerebral blood flow to late recirculation of ischemic brain tissue, are accompanied by changes in neuroinflammation in the brain [13]. Neuroinflammation increases brain damage, leading to the death of surviving neurons in the previously ischemic brain. Neuroinflammation also contributes to the development of glial scars [11,14]. Microglial activation promotes the release of pro-inflammatory cytokines, such as TNF-α, NO, and reactive oxygen species (ROS), which are harmful to the brain tissue after ischemia [15]. Acyl-CoA synthetase long-chain family member 4 is an important isozyme for polyunsaturated fatty acid metabolism and a novel regulator of neuroinflammation in ischemic stroke [16]. Recombinant human fibroblast growth factor 21 treatment promoted functional recovery by modulating microglia/macrophage-mediated neuroinflammation via the nuclear factor kappa B (NF-κB) and peroxisome proliferator-activated receptor gamma signaling pathways in C57BL/6 mice with middle cerebral artery occlusion (MCAO) [17].

Neuronal apoptosis-related molecules: These molecules mainly include the Bcl-2 family, caspase family, and TNF receptor superfamily, which are involved in the regulation of cell apoptosis after brain injury. Downregulation of the TNF-α and TNF receptor 1 proteins can effectively alleviate neurological impairment after cerebral ischemic injury [18,19]. Within hours of an ischemic stroke, neurons can be permanently damaged or die. Therefore, development of treatment options to save damaged neurons is important [20].

Steady-state balance of Ca^2+^: Ca^2+^ and N-methyl-D-aspartate (NMDA) receptors play a central role in regulating excitotoxic neuronal death after cerebral ischemia. Ca^2+^ homeostasis is necessary to maintain normal physiological functions of cells. After cerebral ischemic injury, Ca^2+^ homeostasis is disrupted, leading to Ca^2+^ pump dysfunction, mitochondrial damage, cell membrane depolarization, release of a large amount of glutamate, and NMDA receptor activation, resulting in Ca^2+^ influx and activation of Ca^2+^-dependent protease-induced cell death pathways [21–23].

Oxidative stress: In response to ischemic stroke, excessive ROS can cause substantial cellular injury and vascular effects. Oxidative stress induced by ROS overproduction is associated with the ischemia-reperfusion injury process in ischemic stroke [24,25]. Neuronal apoptosis, inflammatory signaling pathway activation, and blood-brain barrier damage induced by oxidative stress can aggravate neurodegeneration and cell death during ischemic stroke [26–29].

Autophagy: Beclin-1 is an autophagy-associated protein that strongly induces autophagy and plays a key role in regulating the survival of ischemic neurons [30,31]. Some miRNAs can regulate autophagy by regulating changes in the expression of autophagy-related genes. For example, downregulation of the metastasis-associated lung adenocarcinoma transcript 1 (Malat1) lncRNA inhibits Beclin1-dependent autophagy by regulating miR-30a expression, thus reducing nerve cell death in ischemic stroke [32]. The maternal expression gene 3 (MEG3) lncRNA, which is a cell death promoter, is upregulated and interacts physiologically and functionally with p53 to mediate neuron autophagy in ischemic stroke [20].

Energy metabolism disorders: The sudden interruption or reduction of blood flow and oxygen supply to the brain leads to energy depletion or metabolism disorders during ischemic stroke [33,34]. Increased Ca^2+^ and ROS levels lead to mitochondrial dysfunction, which further aggravates oxidative stress [13,19,33] and is associated with upregulation of reduced nicotinamide adenine dinucleotide phosphate (NADPH) diaphorease expression in hippocampal astrocytes during ischemic stroke [33]. Peroxisome proliferator-activated receptor-gamma coactivator (PGC)-1α is a cofactor of peroxisome proliferator-activated receptor family transcription factors that leads to increased expression of mitochondrial fatty acid oxidation genes in ischemic brain tissue [35]. Adenosine 5’-monophosphate-activated protein kinase (AMPK), which functions as a cellular energy sensor, can protect against cerebral ischemic injury by activating catabolic pathways and inactivating ATP-consuming processes [36].

Excessive levels of intracellular free radicals and NO: Superoxide dismutase is an important antioxidant enzyme in brain tissue and can protect the brain by removing excessive free radicals in cells [37]. NO plays a crucial role in information transmission and neuroprotection in the central nervous system, but excessive release of NO, as an active gaseous free radical, can lead to neuron damage [11]. Endogenous NO derived from neuronal NO synthase plays a deleterious role in delayed cerebral neuronal death and ischemic reperfusion damage [38].

Excitatory amino acid toxicity: Glutamate levels in brain tissue increase significantly following cerebral ischemia-reperfusion injury. Glutamate causes excitotoxicity leading to neuronal death [39]. The expression levels of glutamate transporters (glt-1), gap junction proteins (Cx43), and glutamate receptors (AMPA receptors) and Na^+^-K^+^-ATPase activity are related to the anti-excitotoxic effects involved in protection against neuronal death and functional recovery after ischemic stroke [40]. NMDA receptor antagonists can inhibit the release of excitatory amino acids during ischemic stroke [41,42]. Additionally, the neurotoxicity of NO is mainly mediated by its reaction with oxygen free radicals [43].

Blood-brain barrier damage: Aquaporin-4 (AQP4) is one of the most widely expressed molecules in the brain, especially in astrocytes [44]. Downregulation of AQP4, which is involved in water homeostasis in astrocytes, can increase the integrity of the blood-brain barrier and improve cerebral blood flow after transient MCAO [45]. Nitrite may increase cerebral blood flow via its effects on nitrosylation of mitochondrial proteins [46]. AM-36, which is a Na^+^ channel blocker and an antioxidant, has a neuroprotective effect by modulating neutrophil accumulation in ischemic brain tissue [47,48]. Matrix metalloproteinases (MMPs) act mainly on extracellular endopeptides and can digest proteins located outside the cell [49]. MMP-2 and MMP-9 damage the neurovascular unit and promote blood-brain barrier disruption [34,50]. Elevated serum MMP-9 levels during acute ischemic stroke are associated with an increased risk of death and major disability [51].

Formation of glial scars: Astrocytes release vimentin, nestin, IL-1β, monocyte chemotactic protein-1, and glial fibrillary acidic protein, which contribute to the development of reactive gliosis and the formation of glial scars after ischemia [14,52].

## 2. Mechanism by which IPostC triggers endogenous neuroprotection

Acute or delayed cell death after ischemia-reperfusion ultimately leads to the irreversible damage and clinical sequelae observed in stroke patients [53]. IPostC is a temporary, non-lethal ischemia-reperfusion process that protects the primary ischemic organ by producing a non-traumatic ischemic adaptation after ischemia [54]. This process stimulates protective factors, thereby limiting inflammation and delayed cell death [55–57]. IPostC can reduce ischemia-reperfusion injury by regulating a variety of endogenous neuroprotective mechanisms, including reducing ROS production, alleviating inflammatory and cerebral ischemia-reperfusion injury by activating autophagy, inhibiting inflammation of local injured tissues, endoplasmic reticulum stress, and neuronal apoptosis, improving cerebral blood supply, and regulating phosphorylation of the MAPK, protein kinase C, Notch, and PI3K/Akt pathways [58,59]. However, the role of autophagy in IPostC is currently controversial; some research suggests that autophagy eliminates the neuroprotective effect of IPostC [60–62], but other studies have found that autophagy activates the neuroprotective effect mediated by IPostC [30,63]. In the ischemic brain, clearance of damaged mitochondria through autophagy is beneficial for neuronal survival, while nonselective autophagy is harmful [62,64]. Studies have shown that IPostC activates autophagy in rat hearts with ischemia-reperfusion injury and inhibits cardiomyocyte apoptosis [65]. IPostC can activate autophagy to reduce mitochondrial damage and reduce cell death through the AKT/GSK3β pathway and Bcl-2 phosphorylation [63,66]. Recent studies have shown that IPostC is involved in transcription factor EB transnucleation and target gene transcription to promote functional autophagy in cells [67].

IPostC, including in situ ischemic postconditioning (ISPostC) and remote ischemic postconditioning (RIPostC), induces endogenous neuroprotection by producing transient, non-fatal ischemia during reperfusion [68,69]. RIPostC is an intervention that involves repetitive and transient cycles of occlusion and reperfusion of blood flow to a remote organ, such as inducible ischemia of a hind limb, which protects the brain against subsequent ischemic injury. Furthermore, RIPostC is an attractive therapeutic strategy in terms of cost and ease of intervention delivery because it is performed simply by inflating a blood pressure cuff on an arm or leg [70]. ISPostC involves repetitive and transient cycles of occlusion and reperfusion of arterial flow to primary organs. However, the brain is more sensitive and intolerant to ischemia than other organs, and ISPostC may trigger another ischemic event to induce ischemia in brain tissue, which may cause irreversible damage to ischemia-sensitive brain tissue [71]. This drawback has limited the clinical application of ISPostC.

In recent studies, RIPostC has been shown to produce protective effects in the myocardium, kidney, skeletal muscle, and brain after ischemia. Limbs are often used for RIPostC intervention because of their strong ischemia tolerance and because the degree of ischemia can be easily observed [71–73]. IPostC reduced the brain infarct size in a rodent model of acute focal ischemia-reperfusion brain injury [68]. Exosomes secreted by cells can transport signaling molecules to distant cells or organs, thus achieving signal regulation and communication [74]. Previous studies have shown that exosome treatment reduces the cerebral infarction volume, degree of cerebral edema, and expression of NMDA receptors in brain tissues of ischemia-reperfusion injured rats [75]. Exosomes can alleviate the excitotoxicity of glutamate, and organs subjected to RIPostC can secrete exosomes to mitigate glutamate excitotoxicity in the central nervous system [76]. In a rat model involving 100 min of focal brain ischemia, RIPostC could reduce the infarct size when initiated within 30 min of reperfusion [77]. The serum of a limb subjected to RIPostC showed reduced ROS levels and neutrophil activation via the MyD88-TRAF6-MAPK pathway [78]. RIPostC can inhibit ROS production and excessive intracellular Ca^2+^ accumulation to attenuate direct tissue injury and remove a major stimulus that opens the mitochondrial permeability transition pore during reperfusion [79].

The neuroprotective mechanisms of RIPostC are highly complex and have not been fully elucidated. In addition to attenuation of reperfusion injury [80], RIPostC has anti-inflammatory [81], anti-edema [69,73,82], angioneurogenic [83], anti-platelet, and vasodilatory (enhancement of collateral microvascular circulation) effects on ischemic stroke [84,85] that are mediated through numerous neurohumoral chemical messengers, including those released from endothelial-derived exosomes [86]. The neuronal isoform of NO synthase has also been shown to participate in the neuroprotective effect of RIPostC [87]. RIPostC protects the brain against cerebral ischemia-reperfusion injury by upregulating endothelial NO synthase through the PI3K/Akt pathway following focal cerebral ischemia in rats [85,88]. RIPostC effectively improved blood-brain barrier permeability by upregulating MMP-9 expression and downregulating claudin-5 expression in a rat model of hypoperfusion caused by severe carotid stenosis [80,89]. The neuroprotective mechanism of RIPostC is complex, and it often does not involve a single target but results from combined actions on multiple targets and multiple pathways (Table 2) [82]. Therefore, it is of great importance to systematically explore the roles of specific lncRNAs associated with the brain protection produced by RIPostC, which could be used as potential targets for preclinical studies of ischemic stroke.

**Table 2.** The neuroprotective mechanisms of IPostC in ischemic stroke.

Effect on brain	Neuroprotective mechanism	References
Exosomes	Exosomes can alleviate the excitotoxicity of glutamate and reduce the cerebral infarction volume, cerebral edema, and NMDA receptor expression in the ischemic brain.	[74–76]
Limitation of inflammation and delayed cell death	IPostC can **s**timulate protective factors to alleviate inflammation and delayed cell death.	[55–57,80,81]
Attenuation of reperfusion injury	IPostC can reduce ischemia-reperfusion injury by regulating a variety of endogenous neuroprotective mechanisms, including reducing ROS production, alleviating inflammation, activating autophagy, endoplasmic reticulum stress, and neuronal apoptosis, and improving the cerebral blood supply.	[58,59,81,82]
Regulation of autophagy	Autophagy eliminates or activates the neuroprotective effects mediated by IPostC, including regulation of the inflammatory reaction, ROS production, endoplasmic reticulum stress, neuronal apoptosis, and phosphorylation of the MAPK, PKC, Notch, and PI3K/Akt pathways.	[58–63]
Anti-edema effects	IPostC effectively reduces brain edema after reperfusion by inhibiting AQP4 expression.	[69,73,83]
Enhancement of collateral microvascular circulation	RIPostC can enhance collateral microvascular circulation.	[84–86]
Nitric oxide synthase	1. The neuronal isoform of nitric oxide synthase participates in the neuroprotective effect of RIPostC. 2. RIPostC protects the brain against cerebral ischemia-reperfusion injury by upregulating eNOS through the PI3K/Akt pathway.	1. [87,88] 2. [85]
Alleviation of blood brain barrier disruption	RIPostC can effectively improve blood-brain barrier permeability by activating matrix metalloproteinase-9 and inhibiting claudin-5.	[80,89]
Inhibition of ROS production and excessive intracellular Ca^2+^ accumulation	RIPostC can reduce ROS levels and neutrophil activation through the MyD88-TRAF6-MAPK pathway.	[78,79]

IPostC: ischemic postconditioning, NMDA: N-methyl-D-aspartate, ROS: reactive oxygen species, MAPK: mitogen-activated protein kinase, PKC: protein kinase C, PI3K: phosphoinositide 3-kinase, AQP4: aquaporin-4, RIPostC: remote ischemic postconditioning, eNOS: endothelial nitric oxide synthase.

## 3. Effect lncRNAs on ischemic stroke

Non-coding RNAs regulate important cellular and molecular events through a variety of mechanisms and affect the prognosis of ischemic stroke [90,91]. LncRNAs have been demonstrated to be key gene regulators in many diseases [92–94]. LncRNA is a major type of non-coding RNA with a length of more than 200 nucleotides. Studies have found that lncRNAs are involved in a wide range of biological processes, including genome imprinting, transcription, and post-transcriptional regulation [95–97]. LncRNAs can also function as miRNA sponges by competitively binding miRNAs to regulate mRNA expression [98]. LncRNA is closely related to the expression of neighboring mRNA, suggesting that lncRNAs may exert their function by regulating a predicted mRNA target [99,100].

A summary of some well-studied lncRNAs and their effects on a variety of pathophysiological processes in ischemic stroke is presented in Table 3. The antisense non-coding RNA in the INK4 locus (ANRIL) lncRNA was significantly increased in rats with cerebral infarction, and it regulated the caspase recruitment domain 8 gene expression level and promoted angiogenesis and inflammation through the NF-κB and vascular endothelial growth factor (VEGF) signaling pathways [101–103]. Activation of the lncRNA calcium/calmodulin dependent protein kinase II delta associated transcript 1 (C2dat1) increased calcium/calmodulin dependent protein kinase II delta expression in C57BL/6 mice with ischemic brain injury and then activated the NF-κB signaling pathway to inhibit the inflammatory response [91,104] Upregulation of lncRNA ENSG00000226482 expression activated the adipocytokine signaling pathway, resulting in acute ischemic stroke [105].

**Table 3.** LncRNAs associated with ischemic stroke.

LncRNA	Mechanisms in ischemic stroke	References
ANRIL	Regulation of the expression level of the antisense ANRIL lncRNA and promotion of angiogenesis and inflammation through the NF-κB and VEGF signaling pathways.	[101–103]
C2dat1	Activation of C2dat1 lncRNA can increase calcium/calmodulin dependent protein kinase II delta expression and then activate the NF-κB signaling pathway to inhibit the inflammatory response.	[91,104]
H19	The circulating expression level of lncRNA H19 is significantly upregulated in the plasma of stroke patients, OGD/R SH-SY5Y cells, and MCAO/R rat brains.	[106–109]
Malat1	Silencing Malat1 results in increased expression of pro-inflammatory cytokines, and upregulation of Malat1 lncRNA can alleviate ischemic cerebrovascular and brain parenchymal damage. Malat1 lncRNA affects AQP4 expression by competitively binding miR-145 to promote cerebral ischemia-reperfusion injury.	[110] [111]
MEG3	The expression of MEG3 lncRNA is significantly increased in MCAO mouse brain tissue. Inhibition of MEG3 reduces the cerebral infarction volume and degree of cerebral edema. MEG3 lncRNA accelerates the ischemic stroke process by inhibiting miRNA 424–5p to regulate MAPK pathway activation.	[20] [112]
SNHG12	SNHG12 lncRNA targets miR-199a to reduce cell death and inflammation or targets miR-150 to de-repress its target gene, VEGF, in OGD-injured mouse BMECs. SNHG12 lncRNA is upregulated in mouse neuronal cells during ischemia. SNHG12 lncRNA is upregulated at 24 hours of reperfusion in the post-stroke adult mouse cortex.	[114–117]
FosDT	Inhibition of FosDT lncRNA promotes post-stroke neuroprotection via abrogation of REST-mediated gene silencing. FosDT lncRNA is associated with decreased induction of inflammatory genes, decreased apoptosis, mitochondrial dysfunction, and oxidative stress in the ischemic brain.	[86,87] [120]
NEAT1	NEAT1 lncRNA upregulates HOXA1 to alleviate cell apoptosis and promotes cell viability in neonatal mice with hypoxic-ischemic brain damage. NEAT1 knockout can significantly reduce brain injury by inhibiting microglial activation and release of pro-inflammatory cytokines.	[121] [122]
HOTAIR	Silencing of HOTAIR lncRNA improves the recovery of neurological function after ischemic stroke via the miR-148a-3p/KLF6 axis. HOTAIR lncRNA mediates OGD/R-induced cell injury and angiogenesis in an EZH2-dependent manner.	[123,124]
ENSG00000226482 ENSG00000260539	The ENSG00000226482 and ENSG00000260539 lncRNAs are associated with the PPAR signaling pathway and cell adhesion molecules.	[125]
MIAT	Ischemic stroke patients with higher MIAT lncRNA expression levels have a relatively poor prognosis.	[136]
UCA1	A high UCA1 lncRNA expression level is associated with worse accumulated recurrence and survival in ischemic stroke patients.	[138]

IPostC: ischemic postconditioning, lncRNA: long non-coding RNA, ANRIL: antisense non-coding RNA in the INK4 locus, NF-κB: nuclear factor kappa B, VEGF: vascular endothelial growth factor, C2dat1: calcium/calmodulin dependent protein kinase II delta associated transcript 1, OGD/R: oxygen-glucose deprivation/reoxygenation, MCAO: middle cerebral artery occlusion, Malat1: metastasis-associated lung adenocarcinoma transcript 1, AQP4: aquaporin-4, MEG3: maternal expression gene 3, miR: microRNA, MAPK: mitogen-activated protein kinase, SNHG12: small nucleolar RNA host gene 12, FosDT: Fos downstream transcript, NEAT1: nuclear-enriched abundant transcript1, HOXA1: homeoboxA1, HOTAIR: Hox transcript antisense intergenic RNA, EZH2: enhancer of zeste homolog 2, PPAR: peroxisome proliferator-activated receptor, MIAT: myocardial infarction-associated transcript, UCA1: urothelial carcinoma-associated 1, BMECs: brain microvascular endothelial cells.

LncRNA H19 promotes cell death and autophagy in ischemic stroke. The plasma expression level of lncRNA H19 is of potential clinical diagnostic value for stroke, and the circulating lncRNA H19 expression level was significantly upregulated in stroke patients compared with that in healthy controls [106]. LncRNA H19 was upregulated in oxygen-glucose deprivation (OGD)/reoxygenated SH-SY5Y cells and rat brains subjected to MCAO/reperfusion. Inhibition of H19 expression had a significant protective effect against apoptosis and autophagy in SH-SY5Y cells [107–109].

Expression of lncRNA Malat1 was significantly increased in endothelial cells subjected to OGD and brain tissues of MCAO mice. Silencing of Malat1 in cerebral microvascular endothelial cells and ischemic stroke model mice resulted in increased expression of the pro-inflammatory cytokines CMP-1, IL-6, and E-selectin. Upregulation of lncRNA Malat1 can alleviate ischemic cerebrovascular and brain parenchymal damage and has anti-apoptotic and anti-inflammatory effects in the cerebral microvasculature [110]. LncRNA Malat1 is involved in brain tissue damage induced by ischemia-reperfusion injury by affecting AQP4 expression via competitively binding miR-145 in ischemic stroke [111].

LncRNA MEG3 directly binds to the p53 protein at the endogenous RNA position and regulates the expression of lysyl oxidase 12/15. MEG3 expression was significantly increased in OGD-induced HT22 cells and MCAO mouse brain tissue. Inhibition of MEG3 also reduced the cerebral infarction volume and the degree of cerebral edema in MCAO mice [20]. MEG3 accelerates the ischemic stroke process by inhibiting miRNA 424–5p, which targets semaphorin 3A and activates the MAPK pathway [112].

A recent study found that lncRNA 1810034 contributes to protection of the brain against cerebral ischemic stroke and inhibition of neuroinflammation by suppressing the NF-κB pathway in microglial cells [113]. The small nucleolar RNA host gene 12 (SNHG12) lncRNA protects against OGD-induced injury by activating the AMPK-silent information regulator of transcription 1 (Sirtuin 1) pathway via targeting miR-199a to reduce cell death and inflammation in neurons. It can also target miR-150 to de-repress its target gene, VEGF, and promote capillary-like tube formation in OGD-injured mouse bone marrow microvascular endothelial cells [114,115]. SNHG12 was also upregulated in mouse hippocampal cells during ischemia [116]. SNHG12 was upregulated at 24 hours of reperfusion in the post-stroke adult mouse cortex compared with the sham group [117].

Fos downstream transcript (FosDT) is an ischemia-induced lncRNA that preferentially binds to the activated RE1 silencing transcription factor (REST) complex in response to increased cell death and brain damage in ischemic stroke [118]. Knockdown of FosDT resulted in the de-repression of the REST target genes glutamate ionotropic receptor AMPA type subunit 2, NF-κB2, and glutamate ionotropic receptor NMDA type subunit 1, resulting in reduction of the infarct size and improvement of motor function in adult rats with transient MCAO [119]. A recent study found that the improved functional outcome after stroke in rats with knockout of FosDT lncRNA was associated with decreases in inflammatory gene induction, apoptosis, mitochondrial dysfunction, and oxidative stress [120].

The nuclear-enriched abundant transcript1 (NEAT1) lncRNA upregulates homeoboxA1 (HOXA1) to alleviate cell apoptosis and promotes cell viability in neonatal mice with hypoxic-ischemic brain damage [121]. Studies have shown that NEAT1 lncRNA is highly expressed in rats subjected to MCAO, and NEAT1 knockout can significantly reduce brain injury by reducing the number of activated microglia and their release of pro-inflammatory cytokines [122]. The lncRNA Hox transcript antisense intergenic RNA (HOTAIR) can competitively bind miRNA-148a-3p through a competing endogenous RNA mechanism to upregulate Kruppel-like factor 6 (KLF6) expression, inhibit the signal transducer and activator of transcription 3 pathway, promote cell apoptosis and the inflammatory response, and aggravate nerve injury after ischemic stroke [123]. The HOTAIR lncRNA mediates OGD/reoxygenation-induced cell injury and angiogenesis by the HOTAIR/enhancer of zeste homolog 2 axis in human brain microvascular endothelial cells [124]. A recent study found that 228 upregulated and 16 downregulated differentially expressed lncRNAs may be involved in acute cerebral infarction in adult rats. Among them, lncRNA ENSG00000226482 was associated with the peroxisome proliferator-activated receptor signaling pathway, and lncRNA ENSG00000260539, which interacts with cell adhesion molecules, might be a promising indicator for the early diagnosis of acute stroke [125].

The expression characteristics of lncRNAs in the brain tissue of rats with ischemic stroke reveal the key cellular events and various molecular mechanisms of ischemic stroke [99,126,127]. Significant changes in lncRNAs after stroke have a substantial influence on the stability of mRNA expression [128]. LncRNAs can regulate the expression of genes related to ischemic brain injury by interacting with chromatin-modifying proteins [118,119]. Previous studies have shown that lncRNA expression profiles are extensively altered during acute reperfusion after focal cerebral ischemia in adult rats [129–131], and these significantly altered lncRNAs may be related to epigenetic variation after ischemic stroke [132,133].

Exploring the effects of lncRNAs at the systematic expression pattern level in ischemic stroke is of great importance for developing new therapeutic strategies for ischemic stroke. Previous microarray studies have analyzed the lncRNA expression characteristics after ischemia-reperfusion injury and ISPostC. Compared with those in the ischemia-reperfusion injury group, 2292 lncRNAs were upregulated and 1848 lncRNAs were downregulated in the ISPostC group [78]. Our previous study found that after RIPostC intervention in ischemic stroke model rats, the degree of brain edema was alleviated, lncRNA 017702 expression was upregulated, and pro-inflammatory gene expression was downregulated [134]. At present, few studies have examined the regulatory effect of lncRNAs in RIPostC. Some studies have found that lncRNAs may be involved in the neuroprotective effects of IPostC on ischemic stroke, but the effects and mechanisms of lncRNAs in RIPostC after ischemic stroke are still unclear.

## Therapeutic potential of lncRNAs for ischemic stroke

LncRNAs affect various signaling pathways of cellular processes, such as inflammation, cell apoptosis, cell death, autophagy, angiogenesis, and microglial activation. In recent years, studies have shown that some lncRNAs may be regarded as biomarkers for prognosis and diagnosis. Expression of the NEAT1 lncRNA was increased in patients with acute ischemic stroke, and increased NEAT1 expression was correlated with worse recurrence-free survival [135]. A multivariate analysis revealed that ischemic stroke patients with high lncRNA myocardial infarction-associated transcript (MIAT) expression levels had a relatively poor prognosis [136]. A recent study found that compared with atrial fibrillation patients with ischemic stroke, atrial fibrillation patients without ischemic stroke had significantly lower serum ANRIL lncRNA levels. The serum ANRIL level exhibits good predictive value for ischemic stroke in patients with atrial fibrillation, and its increased expression is correlated with worse recurrence-free survival [137]. The urothelial carcinoma-associated 1 (UCA1) lncRNA might serve as a candidate prognostic biomarker for acute ischemic stroke patients. UCA1 expression was elevated in acute ischemic stroke patients with recurrence or death within 2 years compared with that in patients without recurrence or death within 2 years, and a high UCA1 expression level was associated with worse accumulated recurrence-free survival in ischemic stroke patients [138]. A recent study performed a meta-analysis and various bioinformatics analyses to further explore the biological role and molecular mechanisms of lncRNAs to provide a theoretical basis for the prognosis of ischemic stroke [125].

LncRNAs are promising targets for diagnosis, treatment, and prognosis assessment of ischemic stroke. Although some studies have confirmed the regulatory mechanism of single lncRNAs related to nerve injury and repair after ischemic stroke, systematic studies of the role of many lncRNAs in the pathological process of ischemic stroke and their interaction mechanisms have not been performed. Future studies will need to focus on the functional mechanism of the systematic and comprehensive lncRNA regulatory network associated with ischemic stroke and further explore and verify promising therapeutic markers for ischemic stroke. A large amount of work remains in this area. However, the potential clinical implications are important.

## Conclusion

This review summarizes the mechanisms of cerebral injury in ischemic stroke and the neuroprotective effect of IPostC and describes some lncRNAs associated with regulation of ischemic stroke. LncRNAs have high biological value in the diagnosis and prognosis of ischemic stroke and are expected to become new therapeutic targets. Exploring the functions and mechanisms of these lncRNAs will contribute to the development of promising treatment strategies for ischemic stroke.

## References

[1] Mozaffarian D, Mozaffarian D, Benjamin EJ, et al. Heart Disease and Stroke Statistics-2016 Update: a Report From the American Heart Association. Circulation. 2016;133(4):e38–360. 26673558 10.1161/CIR.0000000000000350

[2] Mitsios N, Gaffney J, Kumar P, et al. Pathophysiology of acute ischaemic stroke: an analysis of common signalling mechanisms and identification of new molecular targets. Pathobiology. 2006;73(4):159–175. 17119345 10.1159/000096017

[3] Deb P, Sharma S, Hassan KM. Pathophysiologic mechanisms of acute ischemic stroke: an overview with emphasis on therapeutic significance beyond thrombolysis. Pathophysiology. 2010;17(3):197–218. 20074922 10.1016/j.pathophys.2009.12.001

[4] Gauberti M, De Lizarrondo SM, Vivien D. The “inflammatory penumbra” in ischemic stroke: from clinical data to experimental evidence. Eur Stroke J. 2016;1(1):20–27. 31008264 10.1177/2396987316630249 PMC6301219

[5] Lambertsen KL, Biber K, Finsen B. Inflammatory cytokines in experimental and human stroke. J Cereb Blood Flow and Metab. 2012;32(9):1677–1698. 22739623 10.1038/jcbfm.2012.88 PMC3434626

[6] Wang YS, Li YX, Zhao P, et al. Anti-inflammation Effects of Oxysophoridine on Cerebral Ischemia-Reperfusion Injury in Mice. Inflammation. 2015;38(6):2259–2268. 26178478 10.1007/s10753-015-0211-4

[7] Nia AM, Kalantaripour TP, Basiri M, et al. Nepeta Dschuparensis Bornm Extract Moderates COX-2 and IL-1β Proteins in a Rat Model of Cerebral Ischemia. Iran J Med Sci. 2017;42(2):179–186. 28360444 PMC5366366

[8] Lu WJ, Zeng LL, Wang Y, et al. Blood microRNA-15a Correlates with IL-6, IGF-1 and Acute Cerebral Ischemia. Curr Neurovasc Res. 2018;15(1):63–71. 29557747 10.2174/1567202615666180319143509

[9] Liu X, Zhang CS, Lu C, et al. A conserved motif in JNK/p38-specific MAPK phosphatases as a determinant for JNK1 recognition and inactivation. Nat Commun. 2016;7:10879. 26988444 10.1038/ncomms10879 PMC4802042

[10] Fann DY-W, Lim Y-A, Cheng Y-L, et al. Evidence that NF-κB and MAPK Signaling Promotes NLRP Inflammasome Activation in Neurons Following Ischemic Stroke. Mol Neurobiol. 2018;55(2):1082–1096. 28092085 10.1007/s12035-017-0394-9

[11] Pluta R, Januszewski S, Czuczwar SJ. Neuroinflammation in Post-Ischemic Neurodegeneration of the Brain: friend, Foe, or Both? Int J Mol Sci. 2021;22(9):4405 . 33922467 10.3390/ijms22094405 PMC8122836

[12] Li L, Dong L, Zhao J, et al. Circulating miRNA-3552 as a Potential Biomarker for Ischemic Stroke in Rats. Biomed Res Int. 2020;2020:4501393. 32724801 10.1155/2020/4501393 PMC7381948

[13] Sekeljic V, Bataveljic D, Stamenkovic S, et al. Cellular markers of neuroinflammation and neurogenesis after ischemic brain injury in the long-term survival rat model. Brain Struct Funct. 2012;217(2):411–420. 21706330 10.1007/s00429-011-0336-7

[14] Radenovic L, Nenadic M, Ułamek-Kozioł M, et al. Heterogeneity in brain distribution of activated microglia and astrocytes in a rat ischemic model of Alzheimer’s disease after 2 years of survival. Aging (Albany NY). 2020;12(12):12251–12267. 32501292 10.18632/aging.103411 PMC7343500

[15] Maida CD, Norrito RL, Daidone M, et al. Neuroinflammatory Mechanisms in Ischemic Stroke: focus on Cardioembolic Stroke, Background, and Therapeutic Approaches. Int J Mol Sci. 2020;21(18):6454. 32899616 10.3390/ijms21186454 PMC7555650

[16] Cui Y, Zhang Y, Zhao X, et al. ACSL4 exacerbates ischemic stroke by promoting ferroptosis-induced brain injury and neuroinflammation. Brain Behav Immun. 2021;93:312–321. 33444733 10.1016/j.bbi.2021.01.003

[17] Wang D, Liu F, Zhu L, et al. FGF21 alleviates neuroinflammation following ischemic stroke by modulating the temporal and spatial dynamics of microglia/macrophages. J Neuroinflammation. 2020;17(1):257. 32867781 10.1186/s12974-020-01921-2 PMC7457364

[18] Ouyang YB, Giffard RG. MicroRNAs affect BCL-2 family proteins in the setting of cerebral ischemia. Neurochem Int. 2014;77:2–8. 24373752 10.1016/j.neuint.2013.12.006 PMC4071131

[19] Zhang Y, Li YW, Wang YX, et al. Remifentanil preconditioning alleviating brain damage of cerebral ischemia reperfusion rats by regulating the JNK signal pathway and TNF-α/TNFR1 signal pathway. Mol Biol Rep. 2013;40(12):6997–7006. 24190484 10.1007/s11033-013-2819-5

[20] Yan H, Yuan J, Gao L, et al. Long noncoding RNA MEG3 activation of p53 mediates ischemic neuronal death in stroke. Neuroscience. 2016;337:191–199. 27651151 10.1016/j.neuroscience.2016.09.017

[21] Kumar VS, Gopalakrishnan A, Naziroğlu M, et al. Calcium ion–the key player in cerebral ischemia. Curr Med Chem. 2014;21(18):2065–2075. 24372212 10.2174/0929867321666131228204246

[22] Wu QJ, Tymianski M. Targeting NMDA receptors in stroke: new hope in neuroprotection. Mol Brain. 2018;11(1):15. 29534733 10.1186/s13041-018-0357-8 PMC5851248

[23] Cappelli J, Khacho P, Wang B, et al. Glycine-induced NMDA receptor internalization provides neuroprotection and preserves vasculature following ischemic stroke. iScience. 2022;25(1):103539. 34977503 10.1016/j.isci.2021.103539 PMC8689229

[24] Kahles T, Brandes RP. NADPH oxidases as therapeutic targets in ischemic stroke. Cell Mol Life Sci. 2012;69(14):2345–2363. 22618244 10.1007/s00018-012-1011-8 PMC11114534

[25] Chen H, Yoshioka H, Kim GS, et al. Oxidative stress in ischemic brain damage: mechanisms of cell death and potential molecular targets for neuroprotection. Antioxid Redox Signal. 2011;14(8):1505–1517. 20812869 10.1089/ars.2010.3576 PMC3061196

[26] Kahles T, Luedike P, Endres M, et al. NADPH oxidase plays a central role in blood-brain barrier damage in experimental stroke. Stroke. 2007;38(11):3000–3006. 17916764 10.1161/STROKEAHA.107.489765

[27] Casas AI, Geuss E, Kleikers PWM, et al. NOX4-dependent neuronal autotoxicity and BBB breakdown explain the superior sensitivity of the brain to ischemic damage. Proc Natl Acad Sci USA. 2017;114(46):12315–12320. 29087944 10.1073/pnas.1705034114 PMC5699031

[28] Canty TG Jr.Jr., Boyle EM Jr.Jr., Farr A, et al. Oxidative stress induces NF-kappaB nuclear translocation without degradation of IkappaBalpha. Circulation. 1999;100(19 Suppl):Ii361–4. 10567330 10.1161/01.cir.100.suppl_2.ii-361

[29] Kleinschnitz C, Grund H, Wingler K, et al. Post-stroke inhibition of induced NADPH oxidase type 4 prevents oxidative stress and neurodegeneration. PLoS Biol. 2010;8(9):e1000479. 20877715 10.1371/journal.pbio.1000479 PMC2943442

[30] Su J, Zhang T, Wang K, et al. Autophagy activation contributes to the neuroprotection of remote ischemic perconditioning against focal cerebral ischemia in rats. Neurochem Res. 2014;39(11):2068–2077. 25082119 10.1007/s11064-014-1396-x

[31] Fan YY, Hu WW, Nan F, et al. Postconditioning-induced neuroprotection, mechanisms and applications in cerebral ischemia. Neurochem Int. 2017;107:43–56. 28087295 10.1016/j.neuint.2017.01.006

[32] Wang P, Liang J, Li Y, et al. Down-regulation of miRNA-30a alleviates cerebral ischemic injury through enhancing beclin 1-mediated autophagy. Neurochem Res. 2014;39(7):1279–1291. 24771295 10.1007/s11064-014-1310-6

[33] Su XT, Wang L, Ma SM, et al. Mechanisms of Acupuncture in the Regulation of Oxidative Stress in Treating Ischemic Stroke. Oxid Med Cell Longev. 2020;2020:7875396. 33178387 10.1155/2020/7875396 PMC7644298

[34] Jickling GC, Liu D, Stamova B, et al. Hemorrhagic transformation after ischemic stroke in animals and humans. J Cereb Blood Flow and Metab. 2014;34(2):185–199. 24281743 10.1038/jcbfm.2013.203 PMC3915212

[35] Ham PB 3rd, Raju R. Mitochondrial function in hypoxic ischemic injury and influence of aging. Prog Neurobiol. 2017;157:92–116. 27321753 10.1016/j.pneurobio.2016.06.006 PMC5161736

[36] Jiang S, Li T, Ji T, et al. AMPK: potential Therapeutic Target for Ischemic Stroke. Theranostics. 2018;8(16):4535–4551. 30214637 10.7150/thno.25674 PMC6134933

[37] Si Z, Liu J, Hu K, et al. Effects of thrombolysis within 6 hours on acute cerebral infarction in an improved rat embolic middle cerebral artery occlusion model for ischaemic stroke. J Cell Mol Med. 2019;23(4):2468–2474. 30697923 10.1111/jcmm.14120 PMC6433693

[38] Jayaraj RL, Azimullah S, Beiram R, et al. Neuroinflammation: friend and foe for ischemic stroke. J Neuroinflammation. 2019;16(1):142. 31291966 10.1186/s12974-019-1516-2 PMC6617684

[39] Bonova P, Burda J, Danielisova V, et al. Delayed post-conditioning reduces post-ischemic glutamate level and improves protein synthesis in brain. Neurochem Int. 2013;62(6):854–860. 23454191 10.1016/j.neuint.2013.02.019

[40] You J, Feng L, Xin M, et al. Cerebral Ischemic Postconditioning Plays a Neuroprotective Role through Regulation of Central and Peripheral Glutamate. Biomed Res Int. 2018;2018:6316059. 30112410 10.1155/2018/6316059 PMC6077516

[41] Graham SH, Chen J, Sharp FR, et al. Limiting ischemic injury by inhibition of excitatory amino acid release. J Cereb Blood Flow and Metab. 1993;13(1):88–97. 8093250 10.1038/jcbfm.1993.11

[42] Liu Y, Chu S, Hu Y, et al. Exogenous Adenosine Antagonizes Excitatory Amino Acid Toxicity in Primary Astrocytes. Cell Mol Neurobiol. 2021;41(4):687–704. 32632892 10.1007/s10571-020-00876-5 PMC11448567

[43] Wang Y, Lu S, Chen Y, et al. Smoothened is a therapeutic target for reducing glutamate toxicity in ischemic stroke. Sci Transl Med. 2021;13(610):eaba3444. 34516830 10.1126/scitranslmed.aba3444

[44] Nagelhus EA, Ottersen OP. Physiological roles of aquaporin-4 in brain. Physiol Rev. 2013;93(4):1543–1562. 24137016 10.1152/physrev.00011.2013 PMC3858210

[45] Patabendige A, Singh A, Jenkins S, et al. Astrocyte Activation in Neurovascular Damage and Repair Following Ischaemic Stroke. Int J Mol Sci. 2021;22(8):4280. 33924191 10.3390/ijms22084280 PMC8074612

[46] Hess DC, Khan MB, Kamat P, et al. Conditioning medicine for ischemic and hemorrhagic stroke. Conditioning med. 2021;4(3): 124–129. PMC8372992 34414362

[47] Weston RM, Jarrott B, Ishizuka Y, et al. AM-36 modulates the neutrophil inflammatory response and reduces breakdown of the blood brain barrier after endothelin-1 induced focal brain ischaemia. Br J Pharmacol. 2006;149(6):712–723. 17016500 10.1038/sj.bjp.0706918 PMC2014659

[48] Ruhnau J, Schulze J, Dressel A, et al. Neuroinflammation, and Poststroke Infection: the Multifaceted Role of Neutrophils in Stroke. J Immunol Res. 2017;2017:5140679. 28331857 10.1155/2017/5140679 PMC5346374

[49] Kurzepa J, Kurzepa J, Golab P, et al. The significance of matrix metalloproteinase (MMP)-2 and MMP-9 in the ischemic stroke. Int J Neurosci. 2014;124(10):707–716. 24304146 10.3109/00207454.2013.872102

[50] Zhang H, Xie Q, Hu J. Neuroprotective Effect of Physical Activity in Ischemic Stroke: focus on the Neurovascular Unit. Front Cell Neurosci. 2022;16:860573. 35317197 10.3389/fncel.2022.860573 PMC8934401

[51] Zhong C, Yang J, Xu T, et al. Serum matrix metalloproteinase-9 levels and prognosis of acute ischemic stroke. Neurology. 2017;89(8):805–812. 28747453 10.1212/WNL.0000000000004257 PMC5580861

[52] Wang H, Song G, Chuang H, et al. Portrait of glial scar in neurological diseases. Int J Immunopathol Pharmacol. 2018;31:2058738418801406. 30309271 10.1177/2058738418801406 PMC6187421

[53] Nour M, Scalzo F, Liebeskind DS. Ischemia-reperfusion injury in stroke. Interv Neurol. 2013;1(3–4):185–199. 25187778 10.1159/000353125 PMC4031777

[54] Halkos ME, Kerendi F, Corvera JS, et al. Myocardial protection with postconditioning is not enhanced by ischemic preconditioning. Ann Thorac Surg. 2004;78(3):961–969. 15337028 10.1016/j.athoracsur.2004.03.033

[55] Wang M, Qi DS, Zhou C, et al. Ischemic preconditioning protects the brain against injury via inhibiting CaMKII-nNOS signaling pathway. Brain Res. 2016;1634:140–149. 26794251 10.1016/j.brainres.2016.01.008

[56] Xing B, Chen H, Zhang M, et al. Ischemic postconditioning inhibits apoptosis after focal cerebral ischemia/reperfusion injury in the rat. Stroke. 2008;39(8):2362–2369. 18583563 10.1161/STROKEAHA.107.507939

[57] Cheng Z, Li L, Mo X, et al. Non-invasive remote limb ischemic postconditioning protects rats against focal cerebral ischemia by upregulating STAT3 and reducing apoptosis. Int J Mol Med. 2014;34(4):957–966. 25092271 10.3892/ijmm.2014.1873 PMC4152138

[58] Zhao H, Ren C, Chen X, et al. From rapid to delayed and remote postconditioning: the evolving concept of ischemic postconditioning in brain ischemia. Curr Drug Targets. 2012;13(2):173–187. 22204317 10.2174/138945012799201621 PMC3346695

[59] Zhou M, Xia ZY, Lei SQ, et al. Role of mitophagy regulated by Parkin/DJ-1 in remote ischemic postconditioning-induced mitigation of focal cerebral ischemia-reperfusion. Eur Rev Med Pharmacol Sci. 2015;19(24):4866–4871. 26744879

[60] Gao L, Jiang T, Guo J, et al. Inhibition of autophagy contributes to ischemic postconditioning-induced neuroprotection against focal cerebral ischemia in rats. PloS one. 2012;7(9):e46092. 23029398 10.1371/journal.pone.0046092 PMC3461004

[61] Wang J, Han D, Sun M, et al. A Combination of Remote Ischemic Perconditioning and Cerebral Ischemic Postconditioning Inhibits Autophagy to Attenuate Plasma HMGB1 and Induce Neuroprotection Against Stroke in Rat. J Mol Neurosci. 2016;58(4):424–431. 26852332 10.1007/s12031-016-0724-9

[62] Baek SH, Noh AR, Kim KA, et al. Modulation of mitochondrial function and autophagy mediates carnosine neuroprotection against ischemic brain damage. Stroke. 2014;45(8):2438–2443. 24938837 10.1161/STROKEAHA.114.005183 PMC4211270

[63] Qi ZF, Luo YM, Liu XR, et al. AKT/GSK3β-dependent autophagy contributes to the neuroprotection of limb remote ischemic postconditioning in the transient cerebral ischemic rat model. CNS Neurosci Ther. 2012;18(12):965–973. 23191937 10.1111/cns.12016 PMC6493377

[64] Zhang X, Yan H, Yuan Y, et al. Cerebral ischemia-reperfusion-induced autophagy protects against neuronal injury by mitochondrial clearance. Autophagy. 2013;9(9):1321–1333. 23800795 10.4161/auto.25132

[65] Wei C, Li H, Han L, et al. Activation of autophagy in ischemic postconditioning contributes to cardioprotective effects against ischemia/reperfusion injury in rat hearts. J Cardiovasc Pharmacol. 2013;61(5):416–422. 23364609 10.1097/FJC.0b013e318287d501

[66] Qi Z, Dong W, Shi W, et al. Bcl-2 phosphorylation triggers autophagy switch and reduces mitochondrial damage in limb remote ischemic conditioned rats after ischemic stroke. Transl Stroke Res. 2015;6(3):198–206. 25744447 10.1007/s12975-015-0393-y

[67] Sun Y, Zhang T, Zhang Y, et al. Ischemic Postconditioning Alleviates Cerebral Ischemia-Reperfusion Injury Through Activating Autophagy During Early Reperfusion in Rats. Neurochem Res. 2018;43(9):1826–1840. 30046966 10.1007/s11064-018-2599-3 PMC6096887

[68] Chen G, Thakkar M, Robinson C, et al. Limb Remote Ischemic Conditioning: mechanisms, Anesthetics, and the Potential for Expanding Therapeutic Options. Front Neurol. 2018;9:40. 29467715 10.3389/fneur.2018.00040 PMC5808199

[69] Han D, Sun M, He PP, et al. Ischemic Postconditioning Alleviates Brain Edema After Focal Cerebral Ischemia Reperfusion in Rats Through Down-Regulation of Aquaporin-4. J Mol Neurosci. 2015;56(3):722–729. 25662982 10.1007/s12031-015-0504-y

[70] Weir P, Maguire R, O’Sullivan SE, et al. A meta-analysis of remote ischaemic conditioning in experimental stroke. J Cereb Blood Flow and Metab. 2021;41(1):3–13. 32538284 10.1177/0271678X20924077 PMC7747156

[71] Li CY, Ma W, Liu KP, et al. Advances in intervention methods and brain protection mechanisms of in situ and remote ischemic postconditioning. Metab Brain Dis. 2021;36(1):53–65. 33044640 10.1007/s11011-020-00562-x

[72] Liu C, Yang J, Zhang C, et al. Remote ischemic conditioning reduced cerebral ischemic injury by modulating inflammatory responses and ERK activity in type 2 diabetic mice. Neurochem Int. 2020;135:104690. 31981607 10.1016/j.neuint.2020.104690

[73] Li CY, Ma W, Liu KP, et al. Different ischemic duration and frequency of ischemic postconditioning affect neuroprotection in focal ischemic stroke. J Neurosci Methods. 2020;346:108921. 32888963 10.1016/j.jneumeth.2020.108921

[74] Xiao B, Chai Y, Lv S, et al. Endothelial cell-derived exosomes protect SH-SY5Y nerve cells against ischemia/reperfusion injury. Int J Mol Med. 2017;40(4):1201–1209. 28849073 10.3892/ijmm.2017.3106 PMC5593464

[75] Kalani A, Chaturvedi P, Kamat PK, et al. Curcumin-loaded embryonic stem cell exosomes restored neurovascular unit following ischemia-reperfusion injury. Int J Biochem Cell Biol. 2016;79:360–369. 27594413 10.1016/j.biocel.2016.09.002 PMC5067233

[76] Lee HK, Finniss S, Cazacu S, et al. Mesenchymal stem cells deliver exogenous miRNAs to neural cells and induce their differentiation and glutamate transporter expression. Stem Cells Dev. 2014;23(23):2851–2861. 25036385 10.1089/scd.2014.0146

[77] Ripley AJ, Jeffers MS, McDonald MW, et al. Neuroprotection by Remote Ischemic Conditioning in Rodent Models of Focal Ischemia: a Systematic Review and Meta-Analysis. Transl Stroke Res. 2021;12(3):461–473. 33405011 10.1007/s12975-020-00882-1

[78] Chen G, Zhang J, Sheng M, et al. Serum of limb remote ischemic postconditioning inhibits fMLP-triggered activation and reactive oxygen species releasing of rat neutrophils. Redox Rep. 2021;26(1):176–183. 34663202 10.1080/13510002.2021.1982515 PMC8530488

[79] Sengul D, Sengul I. Connection of reactive oxygen species as an essential factor for the mechanism of phenomena; ischemic preconditioning and postconditioning: come to age or ripening. North Clin Istanb. 2021;8(6):644–649. 35284791 10.14744/nci.2021.78466 PMC8848490

[80] Li J, Hu XS, Zhou FF, et al. Limb remote ischemic postconditioning protects integrity of the blood-brain barrier after stroke. Neural Regen Res. 2018;13(9):1585–1593. 30127119 10.4103/1673-5374.237122 PMC6126140

[81] Doeppner TR, Kaltwasser B, Kuckelkorn U, et al. Systemic Proteasome Inhibition Induces Sustained Post-stroke Neurological Recovery and Neuroprotection via Mechanisms Involving Reversal of Peripheral Immunosuppression and Preservation of Blood-Brain-Barrier Integrity. Mol Neurobiol. 2016;53(9):6332–6341. 26572637 10.1007/s12035-015-9533-3

[82] Landman TRJ, Schoon Y, Warlé MC, et al. Remote Ischemic Conditioning as an Additional Treatment for Acute Ischemic Stroke. Stroke. 2019;50(7):1934–1939. 31154944 10.1161/STROKEAHA.119.025494

[83] Doeppner TR, Zechmeister B, Kaltwasser B, et al. Very Delayed Remote Ischemic Post-conditioning Induces Sustained Neurological Recovery by Mechanisms Involving Enhanced Angioneurogenesis and Peripheral Immunosuppression Reversal. Front Cell Neurosci. 2018;12:383. 30420796 10.3389/fncel.2018.00383 PMC6216109

[84] Abbasi-Habashi S, Jickling GC, Winship IR. Immune Modulation as a Key Mechanism for the Protective Effects of Remote Ischemic Conditioning After Stroke. Front Neurol. 2021;12:746486. 34956045 10.3389/fneur.2021.746486 PMC8695500

[85] Torres-Querol C, Quintana-Luque M, Arque G, et al. Preclinical evidence of remote ischemic conditioning in ischemic stroke, a metanalysis update. Sci Rep. 2021;11(1):23706. 34887465 10.1038/s41598-021-03003-6 PMC8660795

[86] Kitagawa K, Saitoh M, Ishizuka K, et al. Remote Limb Ischemic Conditioning during Cerebral Ischemia Reduces Infarct Size through Enhanced Collateral Circulation in Murine Focal Cerebral Ischemia. J Stroke Cerebrovasc Dis. 2018;27(4):831–838. 29395650 10.1016/j.jstrokecerebrovasdis.2017.09.068

[87] Pignataro G, Esposito E, Sirabella R, et al. nNOS and p-ERK involvement in the neuroprotection exerted by remote postconditioning in rats subjected to transient middle cerebral artery occlusion. Neurobiol Dis. 2013;54:105–114. 23454199 10.1016/j.nbd.2013.02.008

[88] Gulati P, Singh N, Muthuraman A. Pharmacologic evidence for role of endothelial nitric oxide synthase in neuroprotective mechanism of ischemic postconditioning in mice. J Surg Res. 2014;188(1):349–360. 24439135 10.1016/j.jss.2013.12.015

[89] Yang F, Zhang X, Sun Y, et al. Ischemic postconditioning decreases cerebral edema and brain blood barrier disruption caused by relief of carotid stenosis in a rat model of cerebral hypoperfusion. PloS one. 2013;8(2):e57869. 23469092 10.1371/journal.pone.0057869 PMC3585273

[90] Wei N, Xiao L, Xue R, et al. MicroRNA-9 Mediates the Cell Apoptosis by Targeting Bcl2l11 in Ischemic Stroke. Mol Neurobiol. 2016;53(10):6809–6817. 26660116 10.1007/s12035-015-9605-4

[91] Xu Q, Deng F, Xing Z, et al. Long non-coding RNA C2dat1 regulates CaMKIIδ expression to promote neuronal survival through the NF-κB signaling pathway following cerebral ischemia. Cell Death Dis. 2016;7(3):e2173. 27031970 10.1038/cddis.2016.57 PMC4823958

[92] Qureshi IA, Mehler MF. Emerging roles of non-coding RNAs in brain evolution, development, plasticity and disease. Nat Rev Neurosci. 2012;13(8):528–541. 22814587 10.1038/nrn3234 PMC3478095

[93] Schaukowitch K, Kim TK. Emerging epigenetic mechanisms of long non-coding RNAs. Neuroscience. 2014;264:25–38. 24342564 10.1016/j.neuroscience.2013.12.009 PMC3992294

[94] Briggs JA, Wolvetang EJ, Mattick JS, et al. Mechanisms of Long Non-coding RNAs in Mammalian Nervous System Development, Plasticity, Disease, and Evolution. Neuron. 2015;88(5):861–877. 26637795 10.1016/j.neuron.2015.09.045

[95] Shi X, Sun M, Wu Y, et al. Post-transcriptional regulation of long noncoding RNAs in cancer. Tumour Biol. 2015;36(2):503–513. 25618601 10.1007/s13277-015-3106-y

[96] Kornienko AE, Guenzl PM, Barlow DP, et al. Gene regulation by the act of long non-coding RNA transcription. BMC Biol. 2013;11:59. 23721193 10.1186/1741-7007-11-59 PMC3668284

[97] Fatica A, Bozzoni I. Long non-coding RNAs: new players in cell differentiation and development. Nat Rev Genet. 2014;15(1):7–21. 24296535 10.1038/nrg3606

[98] Wang J, Gong C, Maquat LE. Control of myogenesis by rodent SINE-containing lncRNAs. Genes Dev. 2013;27(7):793–804. 23558772 10.1101/gad.212639.112 PMC3639419

[99] Zhao F, Qu Y, Liu J, et al. Microarray Profiling and Co-Expression Network Analysis of LncRNAs and mRNAs in Neonatal Rats Following Hypoxic-ischemic Brain Damage. Sci Rep. 2015;5:13850. 26349411 10.1038/srep13850 PMC4563552

[100] Ren H, Wang G, Chen L, et al. Genome-wide analysis of long non-coding RNAs at early stage of skin pigmentation in goats (*Capra hircus*). BMC Genomics. 2016;17:67. 26785828 10.1186/s12864-016-2365-3 PMC4719336

[101] Zhang B, Wang D, Ji TF, et al. Overexpression of lncRNA ANRIL up-regulates VEGF expression and promotes angiogenesis of diabetes mellitus combined with cerebral infarction by activating NF-κB signaling pathway in a rat model. Oncotarget. 2017;8(10):17347–17359. 28060742 10.18632/oncotarget.14468 PMC5370045

[102] Cao Y. Positive and negative modulation of angiogenesis by VEGFR1 ligands. Sci Signal. 2009;2(59):re1. 19244214 10.1126/scisignal.259re1

[103] Smith EM, Gregg M, Hashemi F, et al. Corticotropin Releasing Factor (CRF) activation of NF-kappaB-directed transcription in leukocytes. Cell Mol Neurobiol. 2006;26(4–6):1021–1036. 16633893 10.1007/s10571-006-9040-1 PMC11520635

[104] Ye J, Das S, Roy A, et al. Ischemic Injury-Induced CaMKIIδ and CaMKIIγ Confer Neuroprotection Through the NF-κB Signaling Pathway. Mol Neurobiol. 2019;56(3):2123–2136. 29992531 10.1007/s12035-018-1198-2 PMC6394630

[105] Li J, Hao M, Yang B, et al. Long non-coding RNAs expression profile and functional analysis of acute ischemic stroke. Medicine. 2020;99(50):e22964. 33327229 10.1097/MD.0000000000022964 PMC7738114

[106] Wang J, Zhao H, Fan Z, et al. Long Noncoding RNA H19 Promotes Neuroinflammation in Ischemic Stroke by Driving Histone Deacetylase 1-Dependent M1 Microglial Polarization. Stroke. 2017;48(8):2211–2221. 28630232 10.1161/STROKEAHA.117.017387

[107] Wang J, Cao B, Han D, et al. Long Non-coding RNA H19 Induces Cerebral Ischemia Reperfusion Injury via Activation of Autophagy. Aging Dis. 2017;8(1):71–84. 28203482 10.14336/AD.2016.0530 PMC5287389

[108] Tao H, Cao W, Yang JJ, et al. Long noncoding RNA H19 controls DUSP5/ERK1/2 axis in cardiac fibroblast proliferation and fibrosis. Cardiovasc pathol. 2016;25(5):381–389. 27318893 10.1016/j.carpath.2016.05.005

[109] Han W, Fu X, Xie J, et al. MiR-26a enhances autophagy to protect against ethanol-induced acute liver injury. J Mol Med. 2015;93(9):1045–1055. 25877859 10.1007/s00109-015-1282-2 PMC4577542

[110] Zhang X, Tang X, Liu K, et al. Long Noncoding RNA Malat1 Regulates Cerebrovascular Pathologies in Ischemic Stroke. J Neurosci. 2017;37(7):1797–1806. 28093478 10.1523/JNEUROSCI.3389-16.2017 PMC5320610

[111] Wang H, Zheng X, Jin J, et al. LncRNA MALAT1 silencing protects against cerebral ischemia-reperfusion injury through miR-145 to regulate AQP4. J Biomed Sci. 2020;27(1):40. 32138732 10.1186/s12929-020-00635-0 PMC7059719

[112] Xiang Y, Zhang Y, Xia Y, et al. LncRNA MEG3 targeting miR-424-5p via MAPK signaling pathway mediates neuronal apoptosis in ischemic stroke. Aging (Albany NY). 2020;12(4):3156–3174. 32065781 10.18632/aging.102790 PMC7066902

[113] Zhang X, Zhu XL, Ji BY, et al. LncRNA-1810034E14Rik reduces microglia activation in experimental ischemic stroke. J Neuroinflammation. 2019;16(1):75. 30961627 10.1186/s12974-019-1464-x PMC6452518

[114] Long FQ, Su QJ, Zhou JX, et al. LncRNA SNHG12 ameliorates brain microvascular endothelial cell injury by targeting miR-199a. Neural Regen Res. 2018;13(11):1919–1926. 30233065 10.4103/1673-5374.238717 PMC6183049

[115] Zhao M, Wang J, Xi X, et al. SNHG12 Promotes Angiogenesis Following Ischemic Stroke via Regulating miR-150/VEGF Pathway. Neuroscience. 2018;390:231–240. 30193860 10.1016/j.neuroscience.2018.08.029

[116] Yin WL, Yin WG, Huang BS, et al. LncRNA SNHG12 inhibits miR-199a to upregulate SIRT1 to attenuate cerebral ischemia/reperfusion injury through activating AMPK signaling pathway. Neurosci Lett. 2019;690:188–195. 30144542 10.1016/j.neulet.2018.08.026

[117] Bhattarai S, Pontarelli F, Prendergast E, et al. Discovery of novel stroke-responsive lncRNAs in the mouse cortex using genome-wide RNA-seq. Neurobiol Dis. 2017;108:204–212. 28855129 10.1016/j.nbd.2017.08.016

[118] Dharap A, Pokrzywa C, Vemuganti R. Increased binding of stroke-induced long non-coding RNAs to the transcriptional corepressors Sin3A and coREST. ASN neuro. 2013;5(4):283–289. 24063527 10.1042/AN20130029 PMC3806319

[119] Mehta SL, Kim T, Vemuganti R. Long Noncoding RNA FosDT Promotes Ischemic Brain Injury by Interacting with REST-Associated Chromatin-Modifying Proteins. J Neurosci. 2015;35(50):16443–16449. 26674869 10.1523/JNEUROSCI.2943-15.2015 PMC4679824

[120] Mehta SL, Chokkalla AK, Kim T, et al. Long Noncoding RNA Fos Downstream Transcript Is Developmentally Dispensable but Vital for Shaping the Poststroke Functional Outcome. Stroke. 2021;52(7):2381–2392. 33940958 10.1161/STROKEAHA.120.033547 PMC8238840

[121] Zhao J, He L, Yin L. lncRNA NEAT1 Binds to MiR-339-5p to Increase HOXA1 and Alleviate Ischemic Brain Damage in Neonatal Mice. Mol Ther Nucleic Acids. 2020;20:117–127. 32163893 10.1016/j.omtn.2020.01.009 PMC7066222

[122] Jin F, Ou W, Wei B, et al. Transcriptome-Wide Analysis to Identify the Inflammatory Role of lncRNA Neat1 in Experimental Ischemic Stroke. J Inflamm Res. 2021;14:2667–2680. 34188516 10.2147/JIR.S315281 PMC8235937

[123] Huang Y, Wang Y, Liu X, et al. Silencing lncRNA HOTAIR improves the recovery of neurological function in ischemic stroke via the miR-148a-3p/KLF6 axis. Brain Res Bull. 2021;176:43–53. 34391823 10.1016/j.brainresbull.2021.08.003

[124] Wang Y, Mao J, Li X, et al. lncRNA HOTAIR mediates OGD/R-induced cell injury and angiogenesis in a EZH2-dependent manner. Exp Ther Med. 2022;23(1):99. 34976141 10.3892/etm.2021.11022 PMC8674968

[125] Fu J, Yu Q, Xiao J, et al. Long noncoding RNA as a biomarker for the prognosis of ischemic stroke: a protocol for meta-analysis and bioinformatics analysis. Medicine (Baltimore). 2021;100(17):e25596. 33907110 10.1097/MD.0000000000025596 PMC8084069

[126] Karner H, Webb CH, Carmona S, et al. Functional Conservation of LncRNA JPX Despite Sequence and Structural Divergence. J Mol Biol. 2020;432(2):283–300. 31518612 10.1016/j.jmb.2019.09.002

[127] Miao W, Yan Y, Bao TH, et al. Ischemic postconditioning exerts neuroprotective effect through negatively regulating PI3K/Akt2 signaling pathway by microRNA-124. Biomed Pharmacothe. 2020;126:109786. 10.1016/j.biopha.2019.109786 32113052

[128] Dharap A, Nakka VP, Vemuganti R. Effect of focal ischemia on long noncoding RNAs. Stroke. 2012;43(10):2800–2802. 22949471 10.1161/STROKEAHA.112.669465 PMC3458128

[129] Della-Morte D, Guadagni F, Palmirotta R, et al. Genetics and genomics of ischemic tolerance: focus on cardiac and cerebral ischemic preconditioning. Pharmacogenomics. 2012;13(15):1741–1757. 23171338 10.2217/pgs.12.157

[130] Zhang J, Yuan L, Zhang X, et al. Altered long non-coding RNA transcriptomic profiles in brain microvascular endothelium after cerebral ischemia. Exp Neurol. 2016;277:162–170. 26746985 10.1016/j.expneurol.2015.12.014 PMC4761283

[131] Liu J, Wang Y, Akamatsu Y, et al. Vascular remodeling after ischemic stroke: mechanisms and therapeutic potentials. Prog Neurobiol. 2014;115:138–156. 24291532 10.1016/j.pneurobio.2013.11.004 PMC4295834

[132] Dhami KS, Churchward MA, Baker GB, et al. Fluoxetine and citalopram decrease microglial release of glutamate and D-serine to promote cortical neuronal viability following ischemic insult. Mol Cell Neurosci. 2013;56:365–374. 23876875 10.1016/j.mcn.2013.07.006

[133] Shin TH, Lee DY, Basith S, et al. Metabolome Changes in Cerebral Ischemia. Cells. 2020;9(7):1630. 32645907 10.3390/cells9071630 PMC7407387

[134] Ma W, Li CY, Zhang SJ, et al. Neuroprotective effects of long noncoding RNAs involved in ischemic postconditioning after ischemic stroke. Neural Regen Res. 2022;17(6):1299–1309. 34782575 10.4103/1673-5374.327346 PMC8643058

[135] Li P, Duan S, Fu A. Long noncoding RNA NEAT1 correlates with higher disease risk, worse disease condition, decreased miR-124 and miR-125a and predicts poor recurrence-free survival of acute ischemic stroke. J Clin Lab Anal. 2020;34(2):e23056. 31721299 10.1002/jcla.23056 PMC7031604

[136] Zhu M, Li N, Luo P, et al. Peripheral Blood Leukocyte Expression of lncRNA MIAT and Its Diagnostic and Prognostic Value in Ischemic Stroke. J Stroke Cerebrovasc Dis. 2018;27(2):326–337. 29030044 10.1016/j.jstrokecerebrovasdis.2017.09.009

[137] Zeng W, Jin J. The correlation of serum long non-coding RNA ANRIL with risk factors, functional outcome, and prognosis in atrial fibrillation patients with ischemic stroke. J Clin Lab Anal. 2020;34(8):e23352. 32358844 10.1002/jcla.23352 PMC7439435

[138] Ren B, Song Z, Chen L, et al. Long non-coding RNA UCA1 correlates with elevated disease severity, Th17 cell proportion, inflammatory cytokines, and worse prognosis in acute ischemic stroke patients. J Clin Lab Anal. 2021;35(3):e23697. 33458871 10.1002/jcla.23697 PMC7957988

[139] Won SJ, Kim JE, Cittolin-Santos GF, et al. Assessment at the single-cell level identifies neuronal glutathione depletion as both a cause and effect of ischemia-reperfusion oxidative stress. J Neurosci. 2015;35(18):7143–7152. 25948264 10.1523/JNEUROSCI.4826-14.2015 PMC4420782

